# 2268. Antibiotic Stewardship Initiative to identify and optimize antibiotic administration hang-time in 7 intensive care units (ICU) hospitals in Latin America

**DOI:** 10.1093/ofid/ofad500.1890

**Published:** 2023-11-27

**Authors:** Christian Pallares, Debra A Goff, Juan Carlos García, Elsa De La Cadena, Wanda Cornistein, Daniela Santonato, Diogo Boldim, Itaivet Toledo, Rodrigo Ahumada, Nicolás Valdebenito, Jorge Chaverri, Paulo Castañeda-Méndez, Elsa Yasmín Vente, Sara María Cobo Viveros, Luis Hercilla, Katty Chong, Vanessa Moreno, María Virginia Villegas

**Affiliations:** Centro Médico Imbanaco de Cali, Cali, Valle del Cauca, Colombia; The Ohio State University, Columbus, Ohio; Universidad El Bosque, Bogotá, Distrito Capital de Bogota, Colombia; Universidad El Bosque, Bogotá, Distrito Capital de Bogota, Colombia; Hospital Universitario Austral, Buenos aires, Buenos Aires, Argentina; Hospital Universitario Austral, Buenos aires, Buenos Aires, Argentina; Clínica LEMC, Sao Paulo, Distrito Federal, Brazil; Clínica LEMC, Sao Paulo, Distrito Federal, Brazil; Hospital Gustavo Fricke, Santiago de Chile, Region Metropolitana, Chile; Hospital Gustavo Fricke, Santiago de Chile, Region Metropolitana, Chile; Hospital Calderón Guardia, San José, San Jose, Costa Rica; Hospital Medica Sur / Hospital San Angel Inn Universidad, Mexico city, Distrito Federal, Mexico; Clínica Imbanaco grupo quirónsalud, Cali, Valle del Cauca, Colombia; Clínica Imbanaco grupo quirónsalud, Cali, Valle del Cauca, Colombia; Hospital Alberto Sabogal Sologuren, Lima, Lima, Peru; Hospital Alberto Sabogal Sologuren, Lima, Lima, Peru; Hospital Alberto Sabogal Sologuren, Lima, Lima, Peru; Universidad El Bosque, Bogotá, Distrito Capital de Bogota, Colombia

## Abstract

**Background:**

Septic shock studies have identified that delay to the initial antimicrobial administration is the strongest predictor of survival. With every hour of delay mortality increases by 7.6%. The time from the written antibiotic order to intravenous administration or “hang-time” can be several hours due to logistics within the hospital. As the optimization of the antibiotic administration involves the participation of a team in the ICU, Antimicrobial Stewardship Programs (ASP) play an important role in reducing this gap. The present study sought to determine the time between the prescription and the infusion of the antibiotic in 7 Latin American ICU that are implementing ASPs.

**Methods:**

Prevalence study between 2021-2022 in 7 hospitals (Argentina, Brazil, Chile, Costa Rica, Peru, Mexico and Colombia). Hang-time was defined as the time elapsed from the written antibiotic order to actual IV administration. Hang-time compliant was defined as antibiotic administration within an elapsed time of 1 h from the written antibiotic order. Any patient with a suspected infection in the ICU (sepsis and/or septic shock secondary to urinary, intra-abdominal, soft tissue infections and pneumonia), who had a first dose of the antibiotic was included. Patients with incomplete medical records or who died before the administration of the first dose were excluded. For the descriptive analysis, the average of the time difference between the formulation versus the administration of the treatment was determined, as well as the adherence to the administration of the antibiotic in the first hour after being formulated.

**Results:**

1207 prescriptions were evaluated. The most common diagnose was sepsis and septic shock from urinary and pulmonary source. Hangtime adherence was 38.5% (22.5%-74.0%). The time range was 2 to 1080 minutes.

HANGTIME RESULTS BY COUNTRY

**Figure:**
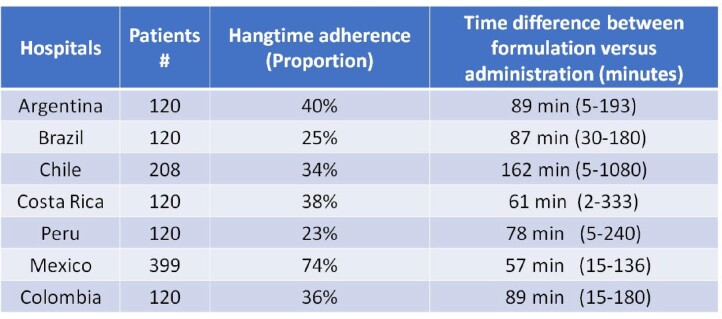

**Conclusion:**

The administration of antibiotics during the first hour in ICU patients was not an adherent practice despite being a risk factor for mortality. A stewardship process improvement protocol with physicians, pharmacists, and nursing collaboration has been developed to improve the hang-time of antibiotics in patients with sepsis. ASPs with limited resources should consider implementing hang-time protocols. Phase 2 will measure compliance of the hang-time protocol and mortality.

**Disclosures:**

**Christian Pallares, MD, MSc**, 3M: Advisor/Consultant|3M: Honoraria|MSD: Advisor/Consultant|MSD: Grant/Research Support|MSD: Honoraria|Pfizer: Advisor/Consultant|Pfizer: Grant/Research Support|Pfizer: Honoraria|Westquímica: Advisor/Consultant|Westquímica: Grant/Research Support|Westquímica: Honoraria **Jorge Chaverri, n/a**, Pfizer: Advisor/Consultant|Pfizer: Honoraria **María Virginia Villegas, n/a**, MSD: Advisor/Consultant|MSD: Grant/Research Support|MSD: Honoraria|Pfizer: Advisor/Consultant|Pfizer: Grant/Research Support|Pfizer: Honoraria|Westquímica: Advisor/Consultant|Westquímica: Grant/Research Support|Westquímica: Honoraria

